# Lipid nanoparticle technologies for the study of G protein-coupled receptors in lipid environments

**DOI:** 10.1007/s12551-020-00775-5

**Published:** 2020-11-19

**Authors:** Steven Lavington, Anthony Watts

**Affiliations:** Biochemistry Department, University of Oxford, South Parks Road, Oxford, OX1 3QU UK

**Keywords:** Nanodisc, rHDL, Lipodisq, SMALP, Lipid-protein interactions, G protein-coupled receptor

## Abstract

G protein-coupled receptors (GPCRs) are a large family of integral membrane proteins which conduct a wide range of biological roles and represent significant drug targets. Most biophysical and structural studies of GPCRs have been conducted on detergent-solubilised receptors, and it is clear that detergents can have detrimental effects on GPCR function. Simultaneously, there is increasing appreciation of roles for specific lipids in modulation of GPCR function. Lipid nanoparticles such as nanodiscs and styrene maleic acid lipid particles (SMALPs) offer opportunities to study integral membrane proteins in lipid environments, in a form that is soluble and amenable to structural and biophysical experiments. Here, we review the application of lipid nanoparticle technologies to the study of GPCRs, assessing the relative merits and limitations of each system. We highlight how these technologies can provide superior platforms to detergents for structural and biophysical studies of GPCRs and inform on roles for protein-lipid interactions in GPCR function.

## Introduction

G protein-coupled receptors (GPCRs) constitute a family of over 800 integral membrane proteins, unified by a shared seven transmembrane helix architecture (Pierce et al. 2002; Fredriksson et al. 2003). Together, GPCRs respond to a diverse array of ligands, including ions, fatty acids, nucleotides and peptides, and their signalling is a component of a great many biological processes. Similarly, GPCR dysfunction is associated with a range of pathologies, including diabetes and obesity (Riddy et al. 2018), cancers (Dorsam and Gutkind 2007) and neurodegenerative diseases (Huang et al. 2017). GPCRs are also valuable drug targets, with a large portion of FDA-approved drugs targeting a relatively small number of receptors (Hauser et al. 2017). There is therefore considerable value in understanding the mechanism of action of these receptors.

Canonically, GPCR signalling involves the binding of ligands to the extracellular portion of a receptor, which in turn promotes signalling through heterotrimeric G proteins, of which there are four subtypes (Strathmann and Gautam 1991; Downes and Gautam 1999). Interactions of GPCRs with arrestins can modulate these signals and produce G protein-independent signals (Reiter and Lefkowitz 2006). Given that a single receptor may signal to multiple G protein subtypes, a great diversity of signalling responses is possible for a single receptor.

There has been tremendous effort in trying to understand the molecular basis of GPCR signalling (reviewed in (Manglik and Kruse 2017; Weis and Kobilka 2018)). Biophysical techniques such as nuclear magnetic resonance and electron paramagnetic resonance spectroscopies have described complex receptor conformational landscapes and how receptor activation relates to these landscapes (Liu et al. 2012; Nygaard et al. 2013; Manglik et al. 2015; Sounier et al. 2015; Ye et al. 2016; Gregorio et al. 2017; Latorraca et al. 2017; Wingler et al. 2019). X-ray crystallography and single-particle cryo-EM have provided a wealth of structural information on a large number of individual GPCRs, with structures available for a range of receptor conformational states and receptor-signalling partner complexes now available (reviewed in (Manglik and Kruse 2017; Weis and Kobilka 2018; García-Nafría and Tate 2019; Wang et al. 2020)).

A majority of studies addressing molecular mechanisms of GPCR signalling make use of receptors solubilised and purified in detergents. GPCRs have evolved to function in the lipid bilayers of biological membranes, and solubilisation of receptors by detergents necessarily disrupts this environment. Detergent solubilisation is often accompanied by a reduction in receptor thermostability (Serrano-Vega et al. 2008; Grisshammer 2009; Lee et al. 2016a) which can lead to loss of receptor activity through denaturation and aggregation. For some GPCRs, this is so severe that they are intractable to study in detergent, and in response to this, technologies to thermostabilise GPCRs through sequence modification have been developed (Tate and Schertler 2009; Dodevski and Plückthun 2011). In addition, detergents may have deleterious effects on the activities of GPCR signalling partners and their interaction with GPCRs; for example, the activity of heterotrimeric G proteins may be significantly perturbed by detergents (Sarvazyan et al. 1998; Kubota et al. 2009). Detergents such as n-dodecyl β-D-maltoside (DDM) and lauryl maltose neopentyl glycol (LMNG) have become popular for solubilising GPCRs (Munk et al. 2019), and there has been progress in developing detergents with even greater preservation of receptor activity (Bae et al. 2016, 2019; Das et al. 2019).

Even if a receptor is sufficiently stable in detergent, the micelle structures adopted by detergents are poor mimics of a lipid bilayer environment. The bilayer environment may modulate GPCR activity through specific lipid contacts and/or through changes in certain physical properties of the bilayer such as lipid dynamics and order, lateral pressure and thickness (reviewed in (Oates and Watts 2011)). Indeed, GPCRs have been shown to interact selectively with specific phospholipids (Watts et al. 1979; Soubias et al. 2006, 2010; Yen et al. 2018), and specific phospholipids can modulate receptor functions such as ligand binding (Oates et al. 2012; Rues et al. 2016; Dawaliby et al. 2016), receptor activation (Dawaliby et al. 2016), G protein binding and coupling (Bubis 1998; Jastrzebska et al. 2009; Inagaki et al. 2012; Dijkman and Watts 2015; Yen et al. 2018; Strohman et al. 2019) and recruitment of GPCR kinases (GRKs) (Komolov et al. 2017) and arrestins (Sommer et al. 2006; Lee et al. 2020; Staus et al. 2020). In addition, GPCR signalling partners may have lipid-binding properties, as has been reported for G proteins (Vögler et al. 2004; Crouthamel et al. 2008; Kosloff et al. 2008; Álvarez et al. 2015), arrestins (Gaidarov et al. 1999; Bayburt et al. 2011; Lally et al. 2017) and GRKs (Onorato et al. 1995; DebBurman et al. 1996; Pitcher et al. 1996).

**Fig. 1:**
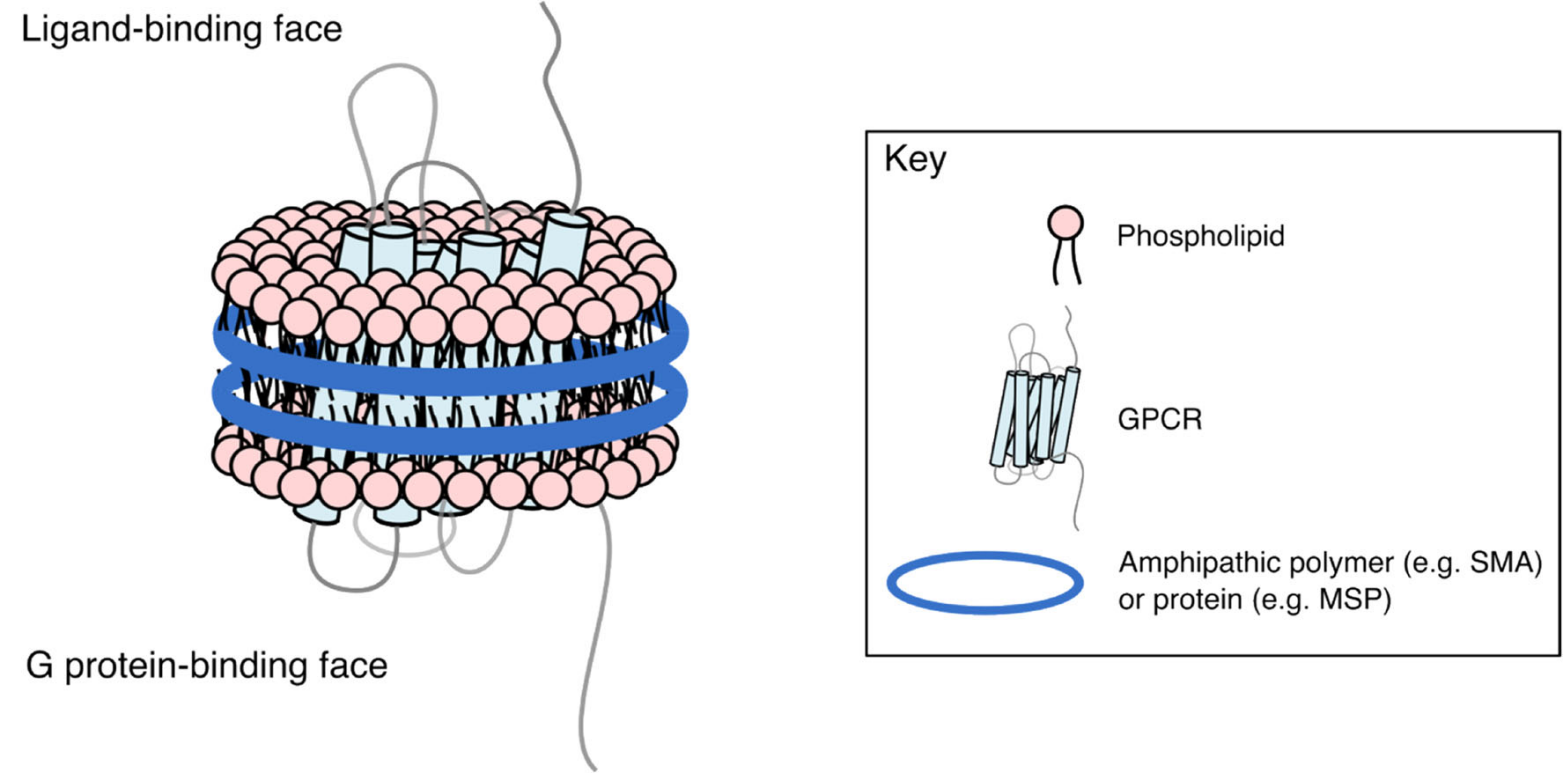
A GPCR lipid nanoparticle. Lipid nanoparticles consist of phospholipids in a discoidal bilayer, solubilised by an amphipathic polymer or protein. Both the ligand-binding face and G protein binding faces of the reconstituted GPCR are accessible to bulk solvent

It is therefore apparent that the absence of a bilayer structure and/or specific lipids and the presence of detergents can have deleterious effects on the behaviour of a GPCR and its signalling partners. In response, membrane mimetic systems have been developed and adapted to study GPCRs in lipidic environments. In particular, lipid nanoparticle systems such as nanodiscs and styrene maleic acid lipid particles (SMALPs) are increasingly used as platforms for biophysical and structural studies of GPCRs. These technologies feature nanoscale (~ 10 nm in diameter) lipid bilayers stabilised by amphipathic protein sequences or synthetic amphipathic polymers (Fig. 1). Their discoidal morphology allows both ligands and intracellular signalling partners such as G proteins access to their binding sites, whilst their small size allows the GPCR lipid nanoparticle to be treated in much the same way as a protein in a detergent micelle.

Here, we will review the application of lipid nanoparticle technologies to the study of GPCRs in lipid environments, addressing their advantages and limitations and highlighting their contribution to our understanding of GPCR signalling and its modulation by lipids.

## Nanodiscs

Nanodiscs are lipid protein nanoparticles consisting of a discoidal lipid bilayer sealed by a ‘belt’ formed by a dimer of membrane scaffold proteins (MSPs) (Bayburt et al. 2002; Denisov and Grinkova 2004). MSPs are amphipathic alpha-helical proteins developed by the Sligar laboratory, derived from the ApoA-1 apolipoprotein of high-density lipoproteins. MSPs with different numbers of helical repeats are available, producing nanoparticles of different diameters (Denisov and Grinkova 2004; Ritchie et al. 2009). In addition to MSPs, nanoscale lipid-protein particles can be formed using wild-type ApoA-1, which can be purified in high levels from serum, but may produce particles less homogeneous in size (Vélez-Ruiz and Sunahara 2011; Denisov and Sligar 2017). The term ‘reconstituted high density lipoprotein’ (rHDL) is sometimes used to refer to lipid nanoparticles formed with ApoA-1 or MSPs. In addition, technologies have been developed using ApoA-1 variants which allow production of circularised nanodiscs (cNDs) with improved stability and decreased dispersity in size (Nasr et al. 2016; Nasr and Wagner 2018). Typically, nanodiscs are formed by incubation of a purified membrane protein with lipids, before addition of MSP and removal of detergent by adsorbent polystyrene beads. The resulting sample can then be purified by size-exclusion chromatography, and empty discs removed by affinity chromatography where necessary (Goddard et al. 2015). Nanodiscs with ~ 9–17 nm diameters can be formed using MSPs (Ritchie et al. 2009), whilst wild-type ApoA-1 can produce nanoparticles of 9–12 nm diameter (Vélez-Ruiz and Sunahara 2011; Dawaliby et al. 2016) and cNDs of up to 80 nm are possible (Nasr and Wagner 2018). The nanodisc system appears to be tolerant of a variety of lipid types (Ritchie et al. 2009) (see also Table 1), such that considerable control over the final lipid composition of the nanodisc bilayer can be exercised.

**Table 1:** Compilation of notable publications utilising nanodiscs/rHDLs to study GPCRs. The type of scaffold protein and lipids used are shown, alongside comments on what the nanodiscs were used for

GPCR	Scaffold protein	Lipid composition	Application	Ref
Neurotensin receptor 1 (NTSR1**)**	MSP1D1	POPG, POPC, POPC/POPG mix (1:1)	Lipid modulation of NTSR1-G_q_ coupling	(Inagaki et al. 2012)
	MSP1E3D1	POPC/POPG mixes (3:1, 1:1)	NTSR1 phosphorylation by GRK2/5	(Inagaki et al. 2015)
	MSP1D1	POPC/POPG (1:1), Brain polar lipids (BPL), POPC/POPG/BPL (1.5:1.1:0.7)	Lipid modulation of NTSR1-Gα_i1_ binding using microscale thermophoresis (MST)	(Dijkman and Watts 2015)
	MSP1D1	POPC/POPG (1:1)	Analysis of NTSR1-Gα_i_ and Gα_s_ binding using surface plasmon resonance (SPR)	(Adamson and Watts 2014)
	cNW9	DMPC/DMPG (3:1)	NMR spectra of ^15^N-labelled receptor ± G_i_ heterotrimer in circularized nanodiscs	(Nasr et al. 2016)
β_1_-adrenergic receptor (β_1_AR)	MSP1E3D1	Various headgroup and acyl chain compositions tested	Effects of nanodisc lipid composition on folding of receptors produced in cell-free system	(Rues et al. 2016)
	Zebrafish apolipoprotein A1 (zap1)	POPC/POPG (7:3)	Structure of a receptor-β-arrestin1 complex by cryo-EM	(Lee et al. 2020)
β_2_-adrenergic receptor (β_2_AR)	MSP1	POPC	Ligand binding and G protein coupling of monomeric receptor	(Leitz et al. 2006)
	Human ApoA-1	POPC/POPG (3:2)	“”	(Whorton et al. 2007)
	Human ApoA-1	POPC/POPG (3:2)	Use of reconstituted receptor to characterise a nanobody, Nb80	(Rasmussen et al. 2011)
	Human ApoA-1	POPC/POPG (3:2)	Use of reconstituted receptor as bait in protein-protein interaction study	(Chung et al. 2013)
	MSP1	POPC/POPS/biotinylated DOPE (2.45:1:0.2)	Single-molecule fluorescence study of receptor activation	(Lamichhane et al. 2015)
	MSP1	POPC/POPG (3:2)	NMR study of receptor conformational dynamics in nanodiscs and detergents	(Kofuku et al. 2014)
	MSP1	POPC/POPG (3:2)	NMR study of conformation of phosphorylated receptor ± β-arrestin1	(Shiraishi et al. 2018)
	Human ApoA-1	DOPC, DOPE, DOPG DOPI and DOPS individually	Lipid modulation of ligand binding and receptor activation	(Dawaliby et al. 2016)
	MSP1E3D1	DOPC, DOPE, DOPG and DOPS individually	Lipid and divalent cation modulation of G protein coupling	(Strohman et al. 2019)
	MSP1D1H5	POPC/POPG (3:2)	Comparison of receptor activity in nanodiscs and detergent	(Staus et al. 2019)
μ-opioid receptor (μOR)	ApoA-1	POPC/POPG (3:2) and BPL/POPC/POPG (1.07:1.5:1)	Ligand binding and G protein coupling of monomeric receptor	(Kuszak et al. 2009)
Adenosine A_2A_-receptor (A_2A_R)	MSP1D1-C9	POPC/POPG (3:2)	Ligand binding of receptor using SPR	(Bocquet et al. 2015)
	MSP1E3	Mixes of lipids with PC-PG headgroups and various acyl chain compositions	Modulation of receptor conformation and G protein coupling by docosahexaenoic acid-containing acyl chains	(Mizumura et al. 2020)
Dopamine D2 receptor (D2R)	MSP1D1	POPC/POPG/Cholesterol (3:2:1)	Cryo-EM structure of receptor-G_i_ complex in a lipid bilayer	(Yin et al. 2020)
Rhodopsin	ApoA-1	POPC/POPG (3:2)	Coupling of monomeric rhodopsin to transducin G protein	(Whorton et al. 2008)
	MSP1E3D1-F1	POPC/POPG (3:2)	Coupling of monomeric rhodopsin to visual arrestin	(Tsukamoto et al. 2010)
	MSP1E3D1-F1	POPC/POPG (3:2)	Rhodopsin photointermediates in nanodiscs mimic those observed in native membranes	(Tsukamoto et al. 2011)
	MSP1E3D1	POPC with varying proportions of POPS	Monomeric rhodopsin is phosphorylated by GRK1 and binds visual arrestin	(Bayburt et al. 2011)
	MSP1E3D1	POPC/POPG (3:2)	Constitutively active rhodopsin mutants are phosphorylated by GRK1, GRK2 and GRK5	(Vishnivetskiy et al. 2013)
	MSP1E3D1	POPC/POPS (7:1)	DEER analysis of active and inactive receptor conformations in detergent and nanodiscs	(Van Eps et al. 2017)
	MSP1E3D1	POPC/POPS (7:1)	DEER analysis of rhodopsin-G_i_ coupling	(Van Eps et al. 2018)
	MSP1E3D1	POPC	Cryo-EM structure of a crosslinked rhodopsin dimer	(Zhao et al. 2019)
Metabotropic glutamate receptor 5 (mGluR5)	MSP1D1	POPC/POPG (3:2)	Structure of inactive-state dimer	(Koehl et al. 2019)
Growth hormone secretagogue receptor (GHSR)	MSP1E3	POPC/POPG/POPS (3:1:1)	Ligand binding, G protein coupling and arrestin recruitment of receptor	(Damian et al. 2012)
	MSP1E3	POPC/POPG/POPS (3:1:1)	Modulation of receptor conformation by ligands and signalling proteins	(Mary et al. 2012)
	MSP1E3	POPC/POPG/POPS (3:1:1)	Analysis of a pre-assembled GHSR-Gq complex	(Damiana et al. 2015)
Parathyroid hormone 1 receptor (PTH1R)	MSP1E3D1	POPC	Production and purification of receptor-nanodiscs immediately after membrane solubilisation	(Mitra et al. 2013)
Glucagon-like peptide-1 receptor (GLP1R)	MSP1E3D1	POPC	“”	(Cai et al. 2017)
Metabotropic glutamate receptor 2 (mGluR2)	MSP1D1	POPC	Analysis of ligand binding and G protein coupling by monomeric and dimeric receptors	(El Moustaine et al. 2012)
M2 muscarinic receptor (M2R)	MSP1E3D1	POPC	G protein coupling of nanodisc-reconstituted receptor versus liposome-reconstituted tetramers	(Redka et al. 2014)
	MSP1E3D1	POPC/POPG	Structure of receptor complex with β-arrestin1	(Staus et al. 2020)
Leukotriene B4 receptor 2 (BLT2R)	MSP1D1	DMPC	Analysis of receptor conformational states by NMR	(Casiraghi et al. 2016)
Neurokinin receptor 1 (NK1R)	Δ49 truncation of ApoA-1	DMPC	Analysis of receptors co-translationally inserted into nanodiscs in a cell-free system	(Gao et al. 2012)
C-C Chemokine receptor 5 (CCR5)	MSP3	POPC, lipids from Sf9 cells	NMR study of ligand-GPCR interactions	(Yoshiura et al. 2010)
C-X Chemokine receptor 1 (CXCR1)	MSP1D1ΔH5	DMPC	""	(Berkamp et al. 2017)

**Fig. 2:**
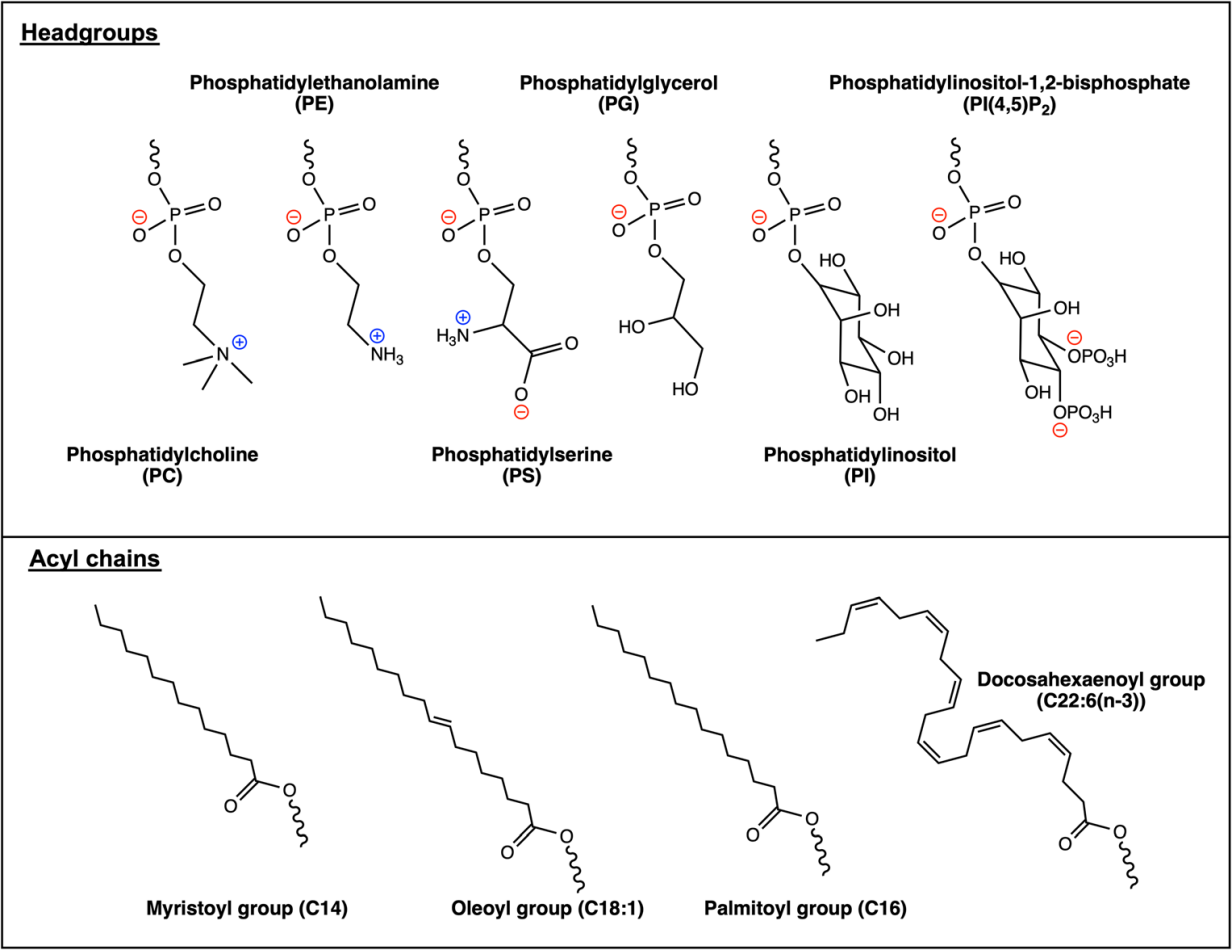
Structures of lipid headgroups and acyl chains. Chemical structures are shown for lipid headgroup and acyl chain structures that have been used to reconstitute GPCRs in nanodiscs

Nanodiscs therefore offer the opportunity to reconstitute a GPCR into a lipid environment of defined lipid composition and size, in which both faces of the GPCR are solvent accessible. The first two demonstrations of nanodisc/rHDL reconstitution of a GPCR employed the β_2_-adrenergic receptor (β_2_AR) (Leitz et al. 2006; Whorton et al. 2007). In a 2006 paper, Sligar and colleagues used MSP1 and the phosphatidylcholine (PC) lipid 1-palmitoyl-2-oleoyl-*sn*-glycero-3-phosphocholine (POPC) (Fig. 2) to reconstitute the receptor, whilst a 2007 paper used the wild-type ApoA-1 protein and a mix of POPC and the phosphatidylglycerol (PG) lipid ﻿1-palmitoyl-2-oleoyl-*sn*-glycero-3-phospho-(1′-*rac*-glycerol) (POPG). In both cases, the monomeric, reconstituted receptor was functional with respect to ligand binding and nucleotide exchange at the G_s_ heterotrimer. Since these initial studies, a wide selection of GPCRs have been reconstituted into nanodiscs and studied by a range of biochemical and biophysical methods (Table 1). Together, the literature demonstrates that it is possible for a GPCR in nanodiscs to bind to ligands, bind and couple to G proteins, recruit GPCR kinases (GRKs) and recruit arrestins (Table 1).

It is generally assumed that the lipid bilayer environment of a nanodisc provides a more native-like environment for the study of GPCRs when compared to detergent micelles, and several reports have directly compared GPCR activity in micelles and nanodiscs. For rhodopsin, the nanodisc-reconstituted receptor activates the transducin G protein heterotrimer to a similar degree to the receptor in native membranes and to a much higher degree than in detergent (Whorton et al. 2007). Moreover, rhodopsin proceeds through similar photointermediates with similar kinetics in both nanodiscs and native membranes, whereas detergent solution perturbs the kinetics of late photointermediates considerably (Tsukamoto et al. 2011). A DEER study of rhodopsin in nanodiscs and detergent reveals that the receptor adopts a more complex conformational ensemble in nanodiscs than in detergent (Van Eps et al. 2017), presumably reflecting a more native-like situation that allows the receptor to couple to multiple signalling partners. For the β_2_–adrenergic receptor (β_2_AR), an NMR study of the deuterated receptor demonstrated greater constitutive activity and slower conformational exchange between receptor active and inactive states in a nanodisc compared to detergents (Kofuku et al. 2014). A recent ^19^F-NMR study similarly found increased constitutive activity for β_2_AR in POPC/POPG nanodiscs compared to detergent and that the receptor in nanodiscs stimulated considerably more GTP turnover at the G_s_ heterotrimer than in detergent (Staus et al. 2019). It is therefore clear that nanodiscs can provide a superior environment for the in vitro study of GPCR signalling when compared to detergents.

Nanodiscs may support higher GPCR activity than detergents because of the absence of inhibitory effects of detergents, the presence of a bilayer structure and/or the presence of specific lipids. Studies on GPCRs in nanodiscs in which lipid composition is varied suggest that specific lipids can have significant modulatory effects on GPCRs in nanodiscs, and so the degree to which native-like receptor activity is supported will depend on nanodisc lipid composition. In a 2016 study, the β_2_AR in ApoA-1 rHDLs containing one of five different lipids was used; acidic PG, phosphatidylserine (PS) and phosphatidylinositol (PI) lipids, and zwitterionic phosphatidylethanolamine (PE) and PC lipids, all with dioleoyl (DO) acyl chains (Dawaliby et al. 2016) (Fig. 2). All five compositions support ligand binding and receptor activation by a nanobody, but acidic phospholipids, especially PG, promote highest ligand binding affinity and augment receptor activation. PE, by contrast, favours an inactive receptor state. In a subsequent study, it was found that nanodiscs containing PG and PS headgroups modulate β_2_AR activation by the G_s_ heterotrimer but surprisingly are inhibitory to β_2_AR activation by the G_i3_ heterotrimer in the absence of divalent cations (Strohman et al. 2019). Instead, PE facilitates β_2_AR activation by G_i3_, demonstrating that one type of lipid can exert different effects at the levels of GPCR conformation and GPCR-G protein coupling. For the neurotensin type 1 receptor (NTSR1), increasing nanodisc PG content increases G_q_ coupling (Inagaki et al. 2012), whilst decreasing amounts of PG and increasing amounts of a PE-rich brain polar lipid mix in a nanodisc increase the affinity of NTSR1 for the Gα_i1_ protein (Dijkman and Watts 2015). For the β_1_AR, an elegant study established which lipid headgroups and acyl chains direct authentic folding of the receptor upon co-translational insertion into a nanodisc in a cell-free expression system (Rues et al. 2016). Consistently, nanodiscs containing lipids with acidic PG or PS headgroups support greater receptor ligand binding activity, whilst short, C14 saturated acyl chains support the least activity and C18 acyl chains with one *trans* double bond support the most activity. For the adenosine A_2A_ receptor (A_2A_R), NMR experiments on the nanodisc-reconstituted receptor were used to demonstrate that polyunsaturated docosahexaenoic acid (DHA) acyl chains shift the receptor conformational ensemble towards states more productive in G protein binding, which correlates with greater observed stimulation of G_s_ by the receptor in nanodiscs containing DHA chains (Mizumura et al. 2020). Taken together, these studies demonstrate the importance of nanodisc lipid composition on observed receptor activity and highlight the power of nanodiscs as a tool to assess specific lipids for modulatory effects on GPCRs.

Recent studies of GPCRs in nanodiscs using cryo-electron microscopy (cryo-EM) demonstrate the advantages of nanodiscs over detergent solutions for structural studies of GPCRs. For both the M2 muscarinic receptor (M2R) and β_1_-adrenergic receptor (β_1_AR), reconstitution into POPC/POPG nanodiscs facilitated coupling to β-arrestin1, which was weak in detergent (Lee et al. 2020; Staus et al. 2020), consistent with previous indications of a role for acidic phospholipids in arrestin recruitment by GPCRs (Gaidarov et al. 1999; Sommer et al. 2006; Tsukamoto et al. 2010). This in turn allowed for structures of complexes of M2R-β-arrestin1 (Staus et al. 2020) and β_1_AR-β-arrestin1 (Lee et al. 2020) to be solved by cryo-EM. In both structures, whilst specific lipid headgroups in the nanodisc are not resolved, it is clear that the C-edge loops of arrestin contact the lipids of the nanodisc, consistent with previous demonstrations of arrestin-lipid interactions by biophysical methods (Lally et al. 2017). Fluorescence experiments on the M2R-β-arrestin1 interaction in nanodiscs confirmed that this arrestin-lipid contact is of significance to receptor-arrestin coupling and subsequent functional outcomes (Staus et al. 2020). Together, these structures demonstrate how a nanodisc can support a feature of GPCR signalling that is poorly reconstituted in detergent and illustrate the protein-lipid contacts that are responsible for this detergent sensitivity. Similarly, a structure of a complex of the dopamine D2 receptor (D2R) and the G_i_ heterotrimer was recently obtained using cryo-EM, with the receptor reconstituted into an MSP1D1 nanodisc containing POPC, POPG and cholesterol (Yin et al. 2020). Whilst structures of GPCR-G_i_ complexes have been obtained in detergents (reviewed in (García-Nafría and Tate 2019; Wang et al. 2020)), this structure demonstrates several interactions between lipids and amino acid residues from both the receptor and G protein. Helix 8 of the receptor is observed buried amongst the headgroup region of the phospholipid bilayer, consistent with biophysical studies (Dijkman et al. 2020) and molecular dynamics simulations (Sensoy and Weinstein 2015). For the G protein, the α-subunit αN helix runs along the nanodisc surface, residues of the γ-subunit make polar interactions and unexpectedly, direct contacts between Gβ and the nanodisc bilayer are also observed. This structure therefore shows how reconstitution of a receptor into a nanodisc provides important lipid context to GPCR structures, even when the overall architecture of GPCR-G_i_ complexes is known.

Nanodiscs can therefore provide a superior environment for biophysical and structural studies of GPCRs compared to detergents and have provided considerable insight into lipid modulation of GPCRs. However, there are several limitations to the technology and practical aspects to consider for successful nanodisc reconstitution of GPCRs.

The primary issue with nanodiscs is that the reconstitution process requires a detergent-solubilised GPCR. As such, any GPCR that is to be reconstituted into a nanodisc must first pass the gauntlet of detergent solubilisation, which may involve the loss of too much receptor material/activity for the reconstitution to be viable. One solution to this issue is to reconstitute the receptor immediately after membrane solubilisation and before purification and then to purify the receptor from other membrane proteins in nanodiscs; this has been demonstrated for two class B GPCRs (Mitra et al. 2013; Cai et al. 2017). This procedure minimises detergent exposure of the receptor, and limits stripping of functionally important lipids from the receptor by detergent, but does require considerable amounts of MSP, since enough scaffold protein must be present to reconstitute both the target protein and all other membrane proteins solubilised from the membrane into nanodiscs. Another approach is to thermostabilise the receptor sequence to allow detergent-solubilisation and reconstitution. This allows for poorly thermostable receptors to be studied in a more native-like environment but with a significantly different sequence.

An additional limitation of nanodiscs is that the presence of MSP/ApoA-1 in the final nanoparticle can interfere with analysis of the reconstituted GPCR. Since the MSP is a protein, it can produce signals in experiments (e.g. UV spectroscopy, circular dichroism and intrinsic tryptophan fluorescence), which may be difficult to separate from those produced by the reconstituted GPCR.

When undertaking nanodisc reconstitution, there are several parameters that can be varied, and it may be laborious to identify conditions that are optimal for both reconstitution efficiency and receptor activity. Firstly, the receptor should be solubilised in a detergent that both retains significant receptor activity and is simple to remove during nanodisc reconstitution. For example, the popular detergent LMNG has a low critical micellar concentration that means it may be difficult to remove completely during reconstitution (Autzen et al. 2019). Secondly, an appropriate MSP construct and lipid composition should be chosen, and then an optimal MSP/lipid ratio should be determined for reconstitution. MSP1(D1) and MSP1E3D1 have been popular choices for GPCRs (Table 1), forming nanodiscs of approximately 9.7 nm and 12.1 nm, respectively (Ritchie et al. 2009). The type of lipid(s) chosen is very important, since, as has been discussed, both lipid acyl chain and headgroup composition can modulate receptor activity significantly. Practically, since nanodisc reconstitution is most efficient near the phase transition temperature of the chosen lipids (Ritchie et al. 2009), it is advantageous to choose lipids with phase transitions at < 4 °C, such that reconstitution can be performed at temperatures which preserve receptor activity. The most popular lipid composition reported to date is a POPC/POPG mix (Table 1). Considering the consensus of literature concerning lipid modulation of GPCRs, the inclusion of an acidic lipid will be appropriate in many cases, since acidic lipids have been demonstrated to promote receptor activation (Dawaliby et al. 2016) and coupling of arrestin (Sommer et al. 2006; Lee et al. 2020; Staus et al. 2020), GRKs (Komolov et al. 2017) and certain G proteins (Inagaki et al. 2012; Strohman et al. 2019). In choosing an acidic lipid, PG appears to be more potent than PS in activating the β_2_AR and is selectively enriched amongst lipids retained by this receptor upon solubilisation (Dawaliby et al. 2016). In addition, acidic phosphatidylinositol(4,5)-bisphosphate (PI(4,5)P_2_) lipids have been shown to play roles in receptor-arrestin interactions (Gaidarov et al. 1999; Huang et al. 2020) and GRK activity (DebBurman et al. 1996). A recent study showed that PI(4,5)P_2_ selectively associates with GPCRs and positively modulates NTSR1-G_i_ coupling (Yen et al. 2018), whilst association of PI(4,5)P_2_ with NTSR1 was observed in a recent cryo-EM structure (Huang et al. 2020). In some cases, inclusion of zwitterionic PE lipids may be appropriate, since they have been implicated in receptor-G_i_ coupling in several cases (Alves et al. 2005; Dijkman and Watts 2015; Strohman et al. 2019) and can provide direct hydrogen bonds to appropriate receptor residues which are stronger than those with water (Sixl and Watts 1985). Another solution to choosing a lipid composition is to use naturally sourced lipid mixes; for example, porcine brain polar lipids have been successfully used for the μOR and NTSR1 (Kuszak et al. 2009; Dijkman and Watts 2015). However, these lipid mixes may have poorly defined compositions and phase behaviour, and some components may not reconstitute into the nanodisc efficiently (Dijkman and Watts 2015).

Finally, nanodiscs are limited by the ‘bottom-up’ nature of the reconstitution process, which requires that the investigator try to reconstruct a functionally supportive lipid composition for the receptor from individual lipid species. It is laborious to exhaustively screen lipids with nanodiscs given the number of possible headgroup and acyl chain structures possible ( ~ 40,000 different lipids are known to date). Even then, lipids that are sufficient to reconstitute/modulate receptor activity in vitro may not necessarily be lipids that act functionally in vivo. It is also apparent that one lipid may have different effects on different aspects of receptor activity; for example, PE favours an inactive receptor conformation but promotes G_i3_ coupling at the β_2_AR. As a result, there is a need for complementary ‘top down’ sources of information on GPCR-lipid interactions.

## Saposin A nanoparticles

An emerging alternative technology to MSP nanodiscs/rHDLs is the ‘Salipro’ system (Frauenfeld et al. 2016). Similar to nanodiscs, the amphipathic moiety that forms the boundary of the lipid nanoparticle is an α-helical protein, saposin A. Unlike MSP, however, there is only a single protein construct used to reconstitute integral membrane proteins of various sizes (Frauenfeld et al. 2016), with saposin A monomers forming a lipid nanoparticle of appropriate size for the membrane protein of interest. To date, the system has seen successful application in membrane protein cryo-EM studies (Du et al. 2020). Using saposin A, a thermostabilised β_1_AR construct was reconstituted with dimyristoyl PC lipids (Chien et al. 2017). The NMR spectrum of the ^13^C-methionine-labelled receptor was sensitive to both ligand and nanobody binding, demonstrating that the receptor retained function in the saposin A nanoparticles. As such, it is clear that this system can be used to study functional GPCRs and may offer a less laborious route to nanoparticle reconstitution than MSP nanodiscs.

## Polymer lipid nanoparticles

**Fig. 3:**
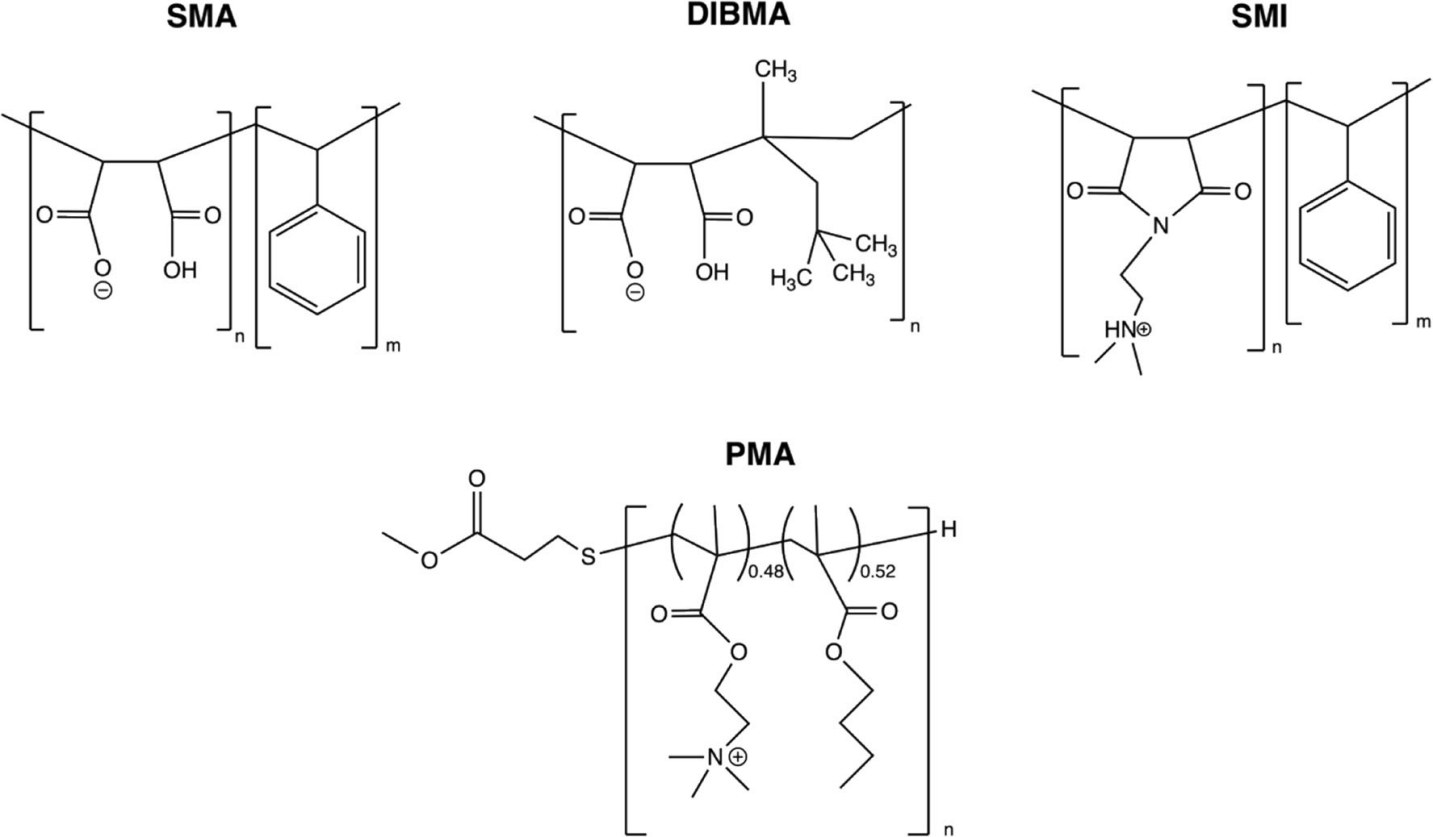
Structures of amphipathic polymers. The structures of SMA, SMI, DIBMA and PMA are shown

The most studied and most frequently used amphipathic polymer for solubilisation and purification of membrane proteins is the styrene-maleic acid co-polymer (SMA), which features hydrophobic styrene and hydrophilic maleic acid moieties that are typically present with gross ratios of 2:1 or 3:1 styrene/maleic acid (Fig. 3). This amphipathic chemical structure allows the polymer to interact with the hydrophobic portion of lipid bilayers and with aqueous solution, in a role analogous to MSP/ApoA-1 in nanodiscs. However, a key difference is that the polymer is able to produce lipid nanoparticles directly from membranes in a process that requires no detergent. This occurs in a multi-step mechanism featuring an initial membrane binding step, followed by polymer insertion into the membrane hydrophobic core and finally solubilisation of the membrane into nanoparticles (Scheidelaar et al. 2015). The resulting nanoparticles are referred to as styrene maleic acid lipid particles (SMALPs) or Lipodisqs (Orwick et al. 2012), are typically ~ 10 nm in diameter (reviewed in (Dörr et al. 2016)) and contain bilayer structures (Jamshad et al. 2015b). The proteins PagP (Knowles et al. 2009) and bacteriorhodopsin (Knowles et al. 2009; Orwick-Rydmark et al. 2012) were the first membrane proteins to be solubilised, purified with SMA and studied using biophysical methods, with both proteins retaining folding and activity in the resulting nanoparticles. Since these initial demonstrations, several studies have been published in which membrane proteins have been isolated directly from the biological membranes of natural sources or heterologous overexpression systems in the complete absence of detergents (reviewed in (Dörr et al. 2016; Bada Juarez et al. 2019)). The resulting particles have been termed ‘native nanodiscs’ (Dörr et al. 2016; Autzen et al. 2019) to distinguish them from cases where polymer lipid nanoparticles are formed from proteoliposomes reconstituted from detergent-solubilised proteins.

Several alternative amphipathic polymers have been applied to and developed for membrane protein studies (reviewed in (Bada Juarez et al. 2019)) (Fig. 3). In most cases, this is in response to well-characterised limitations of the SMA polymer. SMA precipitates at low millimolar concentrations of divalent cations (Dörr et al. 2016; Lee et al. 2016b), absorbs strongly in the far-UV region due to the styrene groups (Oluwole et al. 2017b) and has a limited operating pH range over which it can solubilise membranes (Scheidelaar et al. 2016; Hall et al. 2018). Examples of alternative polymers include DIBMA, which substitutes the styrene moiety for diisobutylene (Oluwole et al. 2017a, b), and SMI, which substitutes maleic acid for a maleimide moiety (Hall et al. 2018), with both polymers being less sensitive to cation-induced precipitation. PMA co-polymers are a third example of alternative polymers and are notable in that their structures are not based on the SMA scaffold (Yasuhara et al. 2017). In all cases, these polymers are capable of producing discoidal bilayers with diameters in the 10–20-nm range and have been used to solubilise and purify specific membrane proteins into nanoparticles from biological membranes (Oluwole et al. 2017a; Barniol-Xicota and Verhelst 2018; Hall et al. 2018; Lavington and Watts 2021).

Since amphipathic polymers can be used to solubilise membrane proteins directly into nanoscale bilayers from biological membranes, they carry considerable potential for the study of GPCRs. Firstly, they negate the need for detergents and their potentially deleterious consequences on receptor stability and function. Secondly, polymers may preserve a lipid environment supportive of receptor function throughout receptor solubilisation and purification and provide opportunities for analysis of this native-like lipid environment. The use of amphipathic polymers to produce GPCR lipid nanoparticles is in its relative infancy when compared to MSP nanodiscs and related technologies. To date, SMA has been used to solubilise several different GPCRs: the A_2A_R (Jamshad et al. 2015a), cannabinoid receptor 1 (CB1R) (Luna et al. 2018), the melatonin-1 receptor (MT_1_R) (Logez et al. 2016), the growth hormone secretagogue receptor (GHSR) (Logez et al. 2016), a heterodimer of the GHSR and dopamine 2 receptors (D_2_R) (Damian et al. 2018) and the dopamine D1 receptor (D_1_R) (Bada Juarez et al. 2020) (Table 2). Of these receptors, the A_2A_R, CB1R, MT_1_R and D_1_R have been purified from biological membranes in the complete absence of detergent using SMA, whilst the GHSR and GHSR-D_2_R complex were solubilised from proteoliposomes.

**Table 2:** Studies utilising amphipathic polymer lipid nanoparticles to study GPCRs. The type of polymer and membrane source used are shown

GPCR	Polymer	Membrane source	Comments	Ref
A_2A_R	SMA 2:1	*Pichia pastoris* HEK293	*P. pastoris* material purified using Ni-NTA chromatography	(Jamshad et al. 2015a; Grime et al. 2020; Routledge et al. 2020)
CB1R	SMA 2:1	Sf9	Material purified by Ni-NTA chromatography	(Luna et al. 2018)
MT_1_R	SMA 2:1	*Pichia pastoris*	Purified using Ni-NTA chromatography and size exclusion chromatography	(Logez et al. 2016)
GHSR	SMA 2:1	Proteoliposomes, reconstituted from GHSR expressed in *E. coli*	Purified using Ni-NTA chromatography	“”
GHSR-D_2_R heterodimer	SMA 2:1	Proteoliposomes reconstituted from GHSR expressed in *E.coli* and D_2_R expressed in *Pichia pastoris*	Purified using multiple affinity tags	(Damian et al. 2018)
D_1_R	SMA 3:1	HEK293	Purified using size exclusion chromatography and Ni-NTA chromatography	(Bada Juarez et al. 2020)
NTSR1	PMA	Sf9	Purified using Ni-NTA and M1-anti FLAG chromatography	(Lavington and Watts 2021)
V_1A_R	SMI	HEK293	No purification	(Hall et al. 2018)
β_2_AR	DIBMA	HEK293	No purification	(Harwood et al. 2020)

In all cases of GPCR solubilisation by SMA, some degree of receptor functionality in SMA lipid-nanoparticles has been demonstrated. For the A_2A_R and D_1_R, radioligand binding assays produced similar ligand binding profiles in both the original membranes and in the SMA-solubilised material (Jamshad et al. 2015a; Bada Juarez et al. 2020). Subsequent publications using A_2A_R-SMALPs have also demonstrated ligand binding using fluorescence correlation spectroscopy (Grime et al. 2020) and conformational changes in the receptor caused by inverse agonist binding (Routledge et al. 2020), although in the latter case limited changes were observed upon agonist binding. For the CB1R, authentic folding was confirmed using a conformationally selective antibody (Luna et al. 2018). For the MT_1_R, the abilities of the receptor in SMALPs to couple to G proteins and recruit arrestin were demonstrated (Logez et al. 2016). For the GHSR, G protein coupling and arrestin recruitment were likewise demonstrated in a SMALP, whilst biophysical techniques revealed a similar ensemble of ligand-dependent conformational states for the receptor whether in a SMALP or in an MSP1E3 nanodisc (Logez et al. 2016). Taken together, these findings suggest that SMALPs provide an environment that supports GPCR function and that solubilisation by SMA does not negatively impact receptor functionality.

GPCRs in SMALPs exhibit improved thermostability compared with GPCRs in detergent solution. A_2A_R-SMALPs from *Pichia pastoris* and HEK293 membranes displayed thermostability increases of 5.5° and 4° compared to DDM, respectively, in addition to resistance to multiple freeze-thaw cycles (Jamshad et al. 2015a). Similarly, the GHSR in SMALPs or MSP nanodiscs retained a high degree of specific radioligand binding over the course of a week, versus a significant loss in binding seen for the DDM receptor (Logez et al. 2016). For the CB1R, an increase in thermostability of ~ 9° was observed for SMALPs compared to DDM detergent, whilst binding to a conformationally sensitive antibody was resistant to multiple freeze-thaw cycles in SMALPs but not in DDM (Luna et al. 2018). Together with demonstrations of receptor function, these results suggest that SMALPs can provide a superior platform for biophysical and structural studies of GPCR function when compared to detergents.

There is also evidence that SMA solubilisation of GPCRs into lipid nanoparticles can preserve protein-protein interactions between GPCR dimers. A heterodimer of GHSR and D2R assembled from purified proteins into proteoliposomes was solubilised using SMA 2:1 (Damian et al. 2018). The resulting particles were purified using tandem chromatography and displayed robust ligand binding and G protein activation at each receptor. Whilst this publication relied on a GPCR-GPCR complex formed in model membranes, it nonetheless demonstrates the potential for SMA to isolate similar complexes from biological membranes. Notably, the authors found that low polymer-to-lipid ratios were required to achieve functional heterodimer solubilisation, and the resulting discs were of ~ 20 nm in diameter. When more typical polymer-to-lipid ratios were used, 10–12 nm sized discs were produced, but these displayed no G protein coupling activity.

In addition to SMA, the SMI, DIBMA and PMA polymers have recently been applied to solubilisation of GPCRs. The acid-compatible SMI polymer was used to solubilise A_2A_R and vasopressin 1a receptors (V_1a_R) from HEK293 membranes (Hall et al. 2018). Both receptors showed specific binding in radioligand binding assays, although material was not purified. DIBMA has recently been used to solubilise the β_2_AR from HEK293 membranes. Whilst the material was not purified, detailed analysis of the stability and activity of the receptor in the resulting nanoparticles showed that receptor ligand binding affinity was similar in DIBMA nanoparticles, membranes and detergent, whilst receptor thermostability was improved by 10° in DIBMA nanoparticles versus detergent (Harwood et al. 2020). We have shown that a polymethacrylate (PMA) co-polymer can be used to solubilise NTSR1 from Sf9 membranes (Lavington and Watts 2021). NTSR1 solubilised with PMA can be purified in the presence of divalent cations, with the resulting material showing stimulation of both G_q_ and G_s_ heterotrimers to a degree that is comparable to higher concentrations of detergent-purified NTSR1.

To date, studies using amphipathic polymers have established that they are a viable means for detergent-free production of functional GPCRs in lipid nanoparticles from yeast, insect and human cell expression systems, which can be analysed by biochemical and biophysical techniques. However, there are a number of practical considerations when using amphipathic polymers for formation of GPCR lipid nanoparticles.

Firstly, as previously discussed, there are well-established limitations of the SMA polymers that may inhibit their application to the solubilisation and purification of GPCRs. Divalent cations such as Mg^2+^ and Ca^2+^ which precipitate SMA may be required in buffers for purification or activity assays of GPCRs and may also directly modulate GPCR activity (Ye et al. 2018; Hu et al. 2019). Whilst it is clear that some GPCRs in SMA lipid nanoparticles have been purified using Ni-NTA resins and size exclusion chromatography, there are also reports of non-specific interactions between SMA and such resins, which may hinder purification of the target GPCR (Qiu et al. 2018; Autzen et al. 2019). Others have reported that the source of SMA may play a role in its effectiveness in solubilisation and purification, with some sources being very heterogeneous (Autzen et al. 2019). However, in spite of these limitations, it is clear that SMA can work well for GPCRs, and so it will be pertinent to test in most cases. In cases where SMA is less appropriate, the development of alternative polymers has circumvented most of the practical issues of SMA. DIBMA and SMI can both solubilise GPCRs with retention of function into nanoparticles less sensitive to the presence of divalent cations, whilst PMA can solubilise and purify functional NTSR1 in the presence of divalent cations. These studies should therefore pave the way for further use of these newer polymers to study GPCRs, and additional polymers have been reported that have yet to be applied to GPCRs (reviewed in (Bada Juarez et al. 2019)). Given that different polymers may produce nanoparticles of different sizes (Bada Juarez et al. 2019) and may solubilise proteins within different optimal experimental conditions (see below), taken together, SMA and newer polymers constitute a toolkit of polymers that an investigator can screen in a manner similar to different detergents (Autzen et al. 2019).

Irrespective of the kind of polymer used, polymer solubilisation of membranes can be very sensitive to factors such as experimental temperature and salt concentration, so some effort is required to determine optimal conditions for solubilisation and subsequent purification. Studies on simplified model membrane systems show that many variables affect SMA solubilisation kinetics, including properties of the membrane bilayer such as fluidity, lateral pressure and charge density, as well as experimental salt concentration, polymer concentration (Scheidelaar et al. 2015) and pH (Scheidelaar et al. 2016). However, the pattern of behaviour of SMA with respect to biological membranes may vary significantly from studies of model membranes. Indeed, a recent study demonstrated that for SMA solubilisation of the KcsA ion channel overexpressed in *Escherichia coli*, the pattern of pH dependence of KcsA solubilisation was quite different to that of model membrane solubilisation at lower polymer concentrations; this is proposed to result from polymer interactions with charged regions of membrane proteins that are absent from model membrane studies (Kopf et al. 2020). Optimal solubilisation of a given membrane will also likely differ for different polymers; for example, the charge properties of PMA are quite distinct from SMA (Fig. 3), and so patterns of salt sensitivity are likely different. Optimal conditions therefore will depend on the polymer chosen, the charge properties of the membrane and the membrane protein being solubilised, and so empirical screening of different salt concentrations, pH values, temperatures and polymer concentrations for each polymer to be tested is highly recommended. It should also be noted that ideal *solubilisation* conditions for a receptor may not translate to optimal *purification* conditions for that receptor, especially if excess free polymer is present (Kopf et al. 2020), and so dialysis/buffer-exchange steps may be required before purification of solubilised material.

Finally, there are still aspects of the physical characteristics of polymer lipid nanoparticles that are unclear. Some studies have shown that rapid exchange of lipids between individual SMA and DIBMA lipid nanoparticles is possible (Hazell et al. 2016; Cuevas Arenas et al. 2017; Danielczak and Keller 2018), suggesting that these particles are rather more dynamic than MSP nanodiscs (Denisov and Sligar 2017) and that polymers do not act as simple ‘cookie cutters’ at the membrane. GPCR-lipid interactions preserved in SMA lipid nanoparticles may not therefore represent the precise lipid environment surrounding the GPCR prior to solubilisation. Given that exchange of lipids within a bilayer is much faster than the observed exchange between bilayers within nanoparticles (Marsh and Watts 1981) and that there is potential for specific association between lipids and membrane proteins (Watts 1993), strong protein-lipid interactions will presumably be preserved during the collisional exchange process, but weaker interactions may be lost (Cuevas Arenas et al. 2017). It is not yet clear whether collisional exchange is a general feature of all types of polymer lipid nanoparticle. SMALPs also have a broader bilayer phase transition than lipid bilayers in a dispersion (Orwick et al. 2012) or indeed in an MSP nanodisc (Denisov et al. 2005), although this effect is less pronounced PMA lipid nanoparticles (Yasuhara et al. 2017) and DIBMA lipid nanoparticles (Oluwole et al. 2017a). In SMALPs, the extent of interaction between lipids and the surrounding polymer is therefore likely higher than between lipid and MSP in a nanodisc. It is also not clear what controls particle diameter in polymer nanoparticles, with a range of particle diameters reported (reviewed in (Dörr et al. 2016)). Relatedly, the number of lipids in a lipid-polymer nanoparticle may be highly variable, with reports of as few as 11 lipids in PagP SMA lipid nanoparticles (Knowles et al. 2009). Indeed, a cryo-EM structure of an alternative complex III supercomplex in an SMA nanoparticle showed a very thin layer of lipid surrounding the protein (Sun et al. 2018), with the boundary of the particle following the shape of the complex, suggesting that under some conditions a true lipid bilayer may not form in an SMA nanoparticle.

## Discussion and perspectives

It should first be noted that despite the advantages of both lipid nanoparticle technologies in providing lipid context to GPCR studies, detergents clearly play an important role in studies of GPCRs. Detergent solubilisation is a necessary step in nanodisc reconstitution, and if a receptor is stable and demonstrates the desired functionality in detergent, then, depending on the question to be addressed, reconstitution into nanodiscs or attempts at detergent-free purification with polymers may be harder to justify. In some cases, if there is prior information regarding functionally important lipids, it may be possible to re-introduce specific lipids in detergent-solubilised form to improve the function of the receptor. Such an approach was successful for a recent cryo-EM structure of the NTSR1-β-arrestin1 complex (Huang et al. 2020), which did not require the use of nanodiscs and in which PI(4,5)P_2_ added during complexing was observed bound to the receptor. Nonetheless, there is increasing appreciation of the importance of the lipid environment in structural and biophysical studies of GPCRs, and as the amount of literature published in this area increases, it is anticipated that detergent studies will be increasingly complemented by studies in lipidic environments such as nanodiscs and polymer lipid nanoparticles.

Nanodiscs and rHDLs represent a well-validated means to study GPCRs in a defined and experimentally tractable lipid environment. This nanodisc environment can support higher receptor activity than detergents and allow for receptor functions that benefit from the absence of detergent, the presence of a bilayer structure, the presence of specific modulatory lipids or a combination of these factors. Nanodiscs have proved an excellent means for assessing the roles of specific lipids on GPCR function and have added considerable detail to the literature on GPCR-lipid interactions. Conversely, the need to reconstitute functionally supportive lipid environments in a bottom-up manner means that we must be cautious to assume that the lipids chosen necessarily reflect important/annular lipids in vivo, and optimisation of nanodisc reconstitution may be laborious. Saposin A nanoparticles may offer a viable alternative to MSP nanodiscs in such cases. Typically, nanodisc reconstitution will be most suitable for receptors that are stable in detergent, and thermostabilisation of the receptor sequence may be required to achieve this. Moving forward, techniques such as native mass spectrometry may be able to provide complementary ‘top-down’ information on GPCR-lipid interactions; a recent study clearly identified lipids that associate with several different GPCRs after solubilisation (Yen et al. 2018), and in theory, the technique can discriminate between very similar lipids bound to a membrane protein (Gault et al. 2016).

Polymer lipid nanoparticles can potentially overcome the disadvantages of nanodiscs, namely, they can be formed without detergents and rely on the receptor itself to organise its lipid environment rather than the experimentalist. Reports to date show at least four kinds of polymer can be used to solubilise functional GPCRs, and the potential advantages of polymer-lipid nanoparticles for studies of GPCRs clearly justify further use of these systems. Firstly, they can potentially allow poorly thermostable receptors to be studied in lipid environments without need for thermostabilising mutations of the receptor sequence, by negating the need for the detergents. Secondly, polymer-lipid nanoparticles can potentially preserve native-like protein-protein and protein-lipid interactions which would otherwise be perturbed by detergents. The demonstration that SMA can solubilise a GPCR heterodimer from liposomes suggests that it may be possible to isolate complexes of GPCRs with other integral or peripheral membrane proteins formed in biological membranes without detergents. Likewise, it is possible to analyse the lipid content of protein-containing polymer lipid nanoparticles (Dörr et al. 2014; Prabudiansyah et al. 2015; Teo et al. 2019), and cryo-EM structures of bacterial membrane proteins in SMA lipid nanoparticles show well-ordered lipid molecules (Qiu et al. 2018; Sun et al. 2018), suggesting polymers may provide a productive route to information on GPCR-lipid interactions. It is probably incorrect to assume that polymers always act like molecular ‘cookie cutters’ that faithfully capture the lipid environment that surrounds a GPCR in a biological membrane, since collisional exchange of lipids for SMA and DIBMA nanoparticles is possible, but it seems likely that even in such cases functionally important lipids are retained. The current practical limitations of the polymer approach over MSP nanodiscs generally stem from the novelty of the technology; the number of publications using polymers to form lipid nanoparticles is rapidly increasing, and it is anticipated that wider adoption of the technology, as well as application of newer polymers, will allow the technology and its application to GPCRs to be optimised.
